# Supplementary material for: Development of Synthetic mRNAs Encoding Split Cytotoxic Proteins for Selective Cell Elimination Based on Specific Protein Detection
Source: Pharmaceutics. 2023 Jan 7;15(1):213. doi: 10.3390/pharmaceutics15010213 (PMC9867180; doi:10.3390/pharmaceutics15010213)
Supplement: Supplementary file 1 [file pharmaceutics-15-00213-s001.zip › pharmaceutics-2037278-supplementary.pdf]

pharmaceutics

MDPI

Supplementary Materials

Development of Synthetic mRNAs Encoding Split Cytotoxic Proteins for Selective Cell Elimination Based on Specific Protein Detection

Kendall Free, Hideyuki Nakanishi and Keiji Itaka

Table S1. List of primers used to prepare template DNAs for in vitro transcription.

Primer Name	Primer Sequence
HNC-542	CAGTGAATTGTAATACGACTCACTATAAGGCGAATTAAGAGAGAAAAGAAGAGTAAGAA-<br>GAAATATAAGACACCGGTCGCCACCATG
HNT-7	CAGTGAATTGTAATACGACTCACTATAAGGCGA
HNT-9	TTTTTTTTTTTTTTTTTTTTTTTTTTTTTTTTTTTTTTTTTTTTTTTTTTTTTTTTTTTTTTTTTTTTTTTTTTTTTTT<br>TTTTTTTTTTTTTTTTTTTTTTTTTTTTTTTTTTTTTTTTTTTTTTTTTTTTTTTCCTACTCAGGCTTTATTCA

Table S2. Full sequences of template DNAs for in vitro transcription.

ID	hBarnase
Template pDNA	pUTR2-hBarnase
Primers	HNC-542, HNT-7 and HNT-9
Features	T7 promoter (for CleanCap AG Reagent): 11-30, Kozak sequence (including start codon): 79-87, hBarnase gene: 88-417, Stop codon: 418-420
Sequence	CAGTGAATTGTAATACGACTCACTATAAGGCGAATTAAGAGAGAAAAGAAGAGTAA-<br>GAA-<br>GAAATATAAGACACCGGTCGCCACCATGGCCCAAGTGATCAACACCTTCGACGGCGTGG<br>CCGACTACCTGCAGACATACCACAAGCTGCCCGACAACTACATCACCAA-<br>GAGCGAGGCCCAGGCTCTCGGATGGGTTGCCTCTAAGGGAAACCTGGCCGATGTGGCCC<br>CTGGCAAGTCTATCGGCGGCGACATCTTCAGCAACAGAGAGGGCAAGCTGCCTGGCAA-<br>GAGCGGCAGAACTTGGAGAGAGGCCGACATCAACTACACCAGCGGCTTCCGGAACAGC<br>GACCGGATCCTGTACAGCAGCGACTGGCTGATCTACAAGACCACCGACCACTAC-<br>CAGAC-<br>CTTCACCAAGATCAGATGAATCTAGACCTTCTGCGGGGCTTGCCTTCTGGCCATGCCCTT<br>CTTCTCTCCCTTGCACCTGTACCTCTTGGTCTTTGAATAAAGCCTGAGTAG-<br>GAAAAAAAAAAAAAAAAAAAAAAAAAAAAAAAAAAAAAAAAAAAAAAAAAAAAAA<br>AAAAAAAAAAAAAAAAAAAAAAAAAAAAAAAAAAAAAAAAAAAAAAAAAAAAAAA<br>AAAAAAAAAAA
ID	hBarnase-21N

Template pDNA	pUTR2-hBarnase-21N
Primers	HNC-542, HNT-7 and HNT-9
Features	T7 promoter (for CleanCap AG Reagent): 11-30, Kozak sequence (including start codon): 79-87, N-terminal split Barnase gene: 88-147, Caged eNpu N-intein gene: 148-714, Anti-eDHFR nanobody Nb113 gene: 721-1122, Stop codon: 1123-1125
Sequence	CAGTGAATTGTAATACGACTCACTATAAGGCGAATTAAGAGAGAAAAGAAGAGTAA-GAA-GAAATATAAGACACCGGTCGCCACCATGGCCCAAGTGATCAACACCTTCGACGGCGTGGCCGACTACCTGCAGACATACCACAAGCTGTGCCTGAGCTACGAGACAGAGATCCTGAC-CGTG-GAATACGGCCTGCTGCCTATCGGCAAGATCGTGGAAAAGCGGATCGAGTGCACCGTGTACAGCGTGGACAACAACGGCAACATCTACACCCAGCCTGTGGCTCAGTGGCAC-GACAGAGGCAAGCAGAAGGTGTTCGAGTACTGCCTGGAAGATGGCAGCCTGATCAGAGCCACCAAGGACCACAAGTTCATGACAGTGGACGGCCAGATGCTGCCCATCAAAGA-GATCTTCCGGCGGAAGCTGGACCTGATGAGAGTGGACAACCTGCCTAATGGCTCTGGCGGCGAGAACCTGTACTTCCAAGGGGAAAATCTCTACTTTCAAGGCGGCAGCGGCGG-CATCGA-GATCGCCACAGAGAAGTATCTGGGCGAGCAGAACGTGTACGACATCGGCGTGGAACGGGACCACAACTTCGCCCTGAAGAACGGCGGCTACTTCCAGGGAATTGAGATTGCCAC-CGA-GAAATACCTCGGGGAACAGAATGTGTATGATATCGGAGTCGAGCGCGATCACAATTTTGCCCTGAAAAATGGCGGATCCCAGGTCCAGCTGCAAGAGTCTGGCGGAGGACTT-GTTCAGGCTGGCGGAAGCCTGAGACTGAGCTGTACTGCCAGCGGCAGAACCTTCAGCAGCTATGCCATGGGCTGGTTCAGACAGACCCCTGGCAAAGAACGCGAG-TTCGTGGCCGCCATTACATGGGGCGGAAGCACAACACTGTACGCCGACTCTGTGAAGGGCAGATTCACCATGAGCCGGGACAACGCCAAGAACACCGTGTACCTG-CAGATGAACAGCCTGAAGCCAGAGGACACCGCCGTGTACTATTGTGCCGCCGATGGCAGCCAGTACAGAAGCACCTACAGCTTCAGAGACAAGCCCGACTACGGCTCTT-GGGGCCAGGGAACACAAGTGACCGTGTCCTCTCACCACCACCATCACCATTGAATCTAGACCTTCTGCGGGGCTTGCCTTCTGGCCATGCCCTTCTTCTCTCCCTTGCACCTGTAC-CTCTT-GGTCTTTGAATAAAGCCTGAGTAGGAAAAAAAAAAAAAAAAAAAAAAAAAAAAAAAAAAAAAAAAAAAAAAAAAAAAAAAAAAAAAAAAAAAAAAAAAAAAAAAAAAAAAAAAAAAAAAAAAAAAAAAAA
ID	hBarnase-21C
Template pDNA	pUTR2-hBarnase-21C
Primers	HNC-542, HNT-7 and HNT-9
Features	T7 promoter (for CleanCap AG Reagent): 11-30, Kozak sequence (including start codon): 79-87, Anti-eDHFR nanobody CA1698 gene: 91-459, Caged eNpu C-intein gene: 466-792, C-terminal split Barnase gene: 793-1065, Stop codon: 1066-1068

Sequence	CAGTGAATTGTAATACGACTCACTATAAGGCGAATTAAGAGAGAAAAGAAGAGTAA-<br>GAA-<br>GAAATATAAGACACCGGTCGCCACCATGGCCCAGGTCCAGCTGCAAGAGTCTGGCGGA<br>GGACTTGTTCAGGCTGGCGGAAGCCTGAGACTGAGCTGTAAAGCCAGCGG-<br>CATCATCTTCAGCGTGTACAAGATGACCTGGTACAGACAGGCCCCTGGCAAAGAGAG<br>AGCTGGTTGCCCTGATCACCACCAACAACAATACCATGACCGTGGACAGCGTGAAGGG-<br>CAGATTCACCATCAGCCGGGACAACGTGCAGAACACCGTGTACCTGGAAATGAACAAT<br>CTGAAGCCCGAGGACACCGCCGTGTACTACTGCAACGCCAATAGAGGACTGGCCGGAC-<br>CTGCCTATTGGGGCCAGGGAACACAAGTGACCGTGTCCTCTCACCACCACCATCACCAT<br>GGATCCGGCGAACAAGAAGTGTTCGAGTACTGCCTGGAAGATGG-<br>CAGCCTGATCAGAGCCAC-<br>CAAGGACCACAAGTTCATGACCGTGGACGGCCAGATGCTGCCCATCGACGAGATCTTCG<br>AGCGCGAGCTGGACCTGATGAGAGTGGACAACCTGCCTAATGGCTCTGGCGGCGA-<br>GAACCTG-<br>TACTTCCAAGGGGAAAATCTCTACTTTCAAGGCGGCAGCGGCGGCATCAAGATCGCCAC<br>AAGAAAGTACCTGGGCAAGCAGAACGTGTACGACATCGGCGTGGAACGGGAC-<br>CACAACTTCGCCCTGAAGAACGGCTTTATCGCCAGCAACTGCCCCGACAACTACATCAC<br>CAAGAGCGAGGCCCAGGCTCTCGGATGGGTTGCCTCTAAGGGAAAC-<br>CTGGCCGATGTGGCCCCTGGCAAGTCTATCGGCGGCGACATCTTCAGCAACAGAGAGGG<br>CAAGCTGCCTGGCAAGAGCGGCAGAACTTGGAGAGAGGCCGACATCAACTACAC-<br>CAGCGGCTTCCGGAACAGCGACCGGATCCTGTACAGCAGCGACTGGCTGATCTACAAGA<br>CCACCGACCACTACCAGACCTTCACCAAGATCAGATGAATCTAGAC-<br>CTTCTGCGGGGCTT-<br>GCCTTCTGGCCATGCCCTTCTTCTCTCCCTTGCACCTGTACCTCTTGGTCTTTGAATAAAGC<br>CTGAGTAG-<br>GAAAAAAAAAAAAAAAAAAAAAAAAAAAAAAAAAAAAAAAAAAAAAAAAAAAAAAAAAAAA<br>AAAAAAAAAAAAAAAAAAAAAAAAAAAAAAAAAAAAAAAAAAAAAAAAAAAAAAAAAAAAA<br>AAAAAAAAAAA
ID	hBarnase-36N
Template pDNA	pUTR2-hBarnase-36N
Primers	HNC-542, HNT-7 and HNT-9
Features	T7 promoter (for CleanCap AG Reagent): 11-30, Kozak sequence (including start codon): 79-87, N-terminal split Barnase gene: 88-192, Caged eNpu N-intein gene: 193-759, Anti-eDHFR nanobody Nb113 gene: 766-1167, Stop codon: 1168-1170
Sequence	CAGTGAATTGTAATACGACTCACTATAAGGCGAATTAAGAGAGAAAAGAAGAGTAA-<br>GAA-<br>GAAATATAAGACACCGGTCGCCACCATGGCCCAAGTGATCAACACCTTCGACGGCGTGG<br>CCGACTACCTGCAGACATACCACAAGCTGCCCGACAACTACATCACCAA-<br>GAGCGAGGCCCAGGCTCTCGGATGGTGCCTGAGCTACGAGACAGAGATCCTGACCGTGG<br>AATACGGCCTGCTGCCTATCGGCAAGATCGTGGAAAAGCGGATCGAGTGCACCGTG-<br>TACAGCGTGGACAACAACGGCAACATCTACACCCAGCCTGTGGCTCAGTGGCACGACA

	GAGGCAAGCAGAAGGTGTTCGAGTACTGCCTGGAAGATGGCAGCCTGATCAGAGCCAC-CAAGGACCACAAGTTCATGACAGTGGACGGCCAGATGCTGCCCATCAAAGAGATCTTCCGGCGGAAGCTGGACCTGATGAGAGTGGACAACCTGCCTAATGGCTCTGGCGGCGA-GAACCTG-TACTTCCAAGGGGAAAATCTCTACTTTCAAGGCGGCAGCGGCGGCATCGAGATCGCCACAGAGAAGTATCTGGGCGAGCAGAACGTGTACGACATCGGCGTGGAACGGGAC-CACAACTTCGCCCTGAAGAACGGCGGCTACTTCCAGGGAATTGAGATTGCCACCGAGAAATACCTCGGGGAACAGAATGTGTATGATATCGGAGTCGAGCGCGATCACAATTTT-GCCCTGAAAAATGGCGGATCCCAGGTCCAGCTGCAAGAGTCTGGCGGAGGACTTGTTCAGGCTGGCGGAAGCCTGAGACTGAGCTGTACTGCCAGCGGCAGAACCTTCAGCAGC-TATGCCATGGGCTGGTTCAGACAGACCCCTGGCAAAGAACGCGAGTTCGTGGCCGCCATTACATGGGGCGGAAGCACAACACTGTACGCCGACTCTGTGAAGGGCAGATTCAC-CATGAGCCGGGACAACGCCAAGAACACCGTGTACCTGCAGATGAACAGCCTGAAGCCAGAGGACACCGCCGTGTACTATTGTGCCGCCGATGGCAGCCAGTACAGAAGCAC-CTACAGCTTCAGAGACAAGCCCGACTACGGCTCTTGGGGCCAGGGAACACAAGTGACCGTGTCCTCTCACCACCACCATCACCATTGAATCTAGACCTTCTGCGGGGCTT-GCCTTCTGGCCATGCCCTTCTTCTCCCTTGCACCTGTACCTCTTGGTCTTTGAATAAAGCCTGAGTAG-GAAAAAAAAAAAAAAAAAAAAAAAAAAAAAAAAAAAAAAAAAAAAAAAAAAAAAAAAAAAAAAAAAAAAAAAAAAAAAAAAAAAAAAAAAAAAAAAAAAAAAAAAAAAAAAAAAAAAAA
ID	hBarnase-36C
Template pDNA	pUTR2-hBarnase-36C
Primers	HNC-542, HNT-7 and HNT-9
Features	T7 promoter (for CleanCap AG Reagent): 11-30, Kozak sequence (including start codon): 79-87, Anti-eDHFR nanobody CA1698 gene: 91-459, Caged eNpu C-intein gene: 466-792, C-terminal split Barnase gene: 793-1020, Stop codon: 1021-1023
Sequence	CAGTGAATTGTAATACGACTCACTATAAGGCGAATTAAGAGAGAAAAGAAGAGTAA-GAA-GAAATATAAGACACCGGTCGCCACCATGGCCCAGGTCCAGCTGCAAGAGTCTGGCGGAGGACTTGTTCAGGCTGGCGGAAGCCTGAGACTGAGCTGTAAAGCCAGCGG-CATCATCTTCAGCGTGTACAAGATGACCTGGTACAGACAGGCCCCTGGCAAAGAGAGAGAGCTGGTTGCCCTGATCACCACCAACAACAATACCATGACCGTGGACAGCGTGAAGGG-CAGATTCACCATCAGCCGGGACAACGTGCAGAACACCGTGTACCTGGAAATGAACAATCTGAAGCCCGAGGACACCGCCGTGTACTACTGCAACGCCAATAGAGGACTGGCCGGAC-CTGCCTATTGGGGCCAGGGAACACAAGTGACCGTGTCCTCTCACCACCACCATCACCATGGATCCGGCGAACAAGAAGTGTTCGAGTACTGCCTGGAAGATGG-CAGCCTGATCAGAGCCAC-CAAGGACCACAAGTTCATGACCGTGGACGGCCAGATGCTGCCCATCGACGAGATCTTCG

	AGCGCGAGCTGGACCTGATGAGAGTGGACAACCTGCCTAATGGCTCTGGCGGCGA-GAACCTG-TACTTCCAAGGGGAAAATCTCTACTTTCAAGGCGGCAGCGGCGGCATCAAGATCGCCACAAGAAAGTACCTGGGCAAGCAGAACGTGTACGACATCGGCGTGGAACGGGAC-CACAACTTCGCCCTGAAGAACGGCTTTATCGCCAGCAACTGCGTTGCCTCTAAGGGAAACCTGGCCGATGTGGCCCCTGGCAAGTCTATCGGCGGCGACATCTTCAGCAACAGA-GAGGG-CAAGCTGCCTGGCAAGAGCGGCAGAACTTGGAGAGAGGCCGACATCAACTACACCAGCGGCTTCCGGAACAGCGACCGGATCCTGTACAGCAGCGACTGGCTGATCTACAAGAC-CAC-CGACCACTACCAGACCTTCACCAAGATCAGATGAATCTAGACCTTCTGCGGGGCTTGCCTTCTGGCCATGCCCTTCTTCTCTCCCTTGCACCTGTACCTCTTGGTCTTTGAATAAA-GCCTGAGTAGGAAAAAAAAAAAAAAAAAAAAAAAAAAAAAAAAAAAAAAAAAAAAAAAAAAAAAAAAAAAAAAAAAAAAAAAAAAAAAAAAAAAAAAAAAAAAAAAAAAAAAAAAAAAAAAAAAAAA
ID	hBarnase-65N
Template pDNA	pUTR2-hBarnase-65N
Primers	HNC-542, HNT-7 and HNT-9
Features	T7 promoter (for CleanCap AG Reagent): 11-30, Kozak sequence (including start codon): 79-87, N-terminal split Barnase gene: 88-279, Caged eNpu N-intein gene: 280-846, Anti-eDHFR nanobody Nb113 gene: 853-1254, Stop codon: 1255-1257
Sequence	CAGTGAATTGTAATACGACTCACTATAAGGCGAATTAAGAGAGAAAAGAAGAGTAA-GAA-GAAATATAAGACACCGGTCGCCACCATGGCCCAAGTGATCAACACCTTCGACGGCGTGGCCGACTACCTGCAGACATACCACAAGCTGCCCGACAACTACATCACCAA-GAGCGAGGCCCAGGCTCTCGGATGGGTTGCCTCTAAGGGAAACCTGGCCGATGTGGCCCCTGGCAAGTCTATCGGCGGCGACATCTTCAGCAACAGAGAGGGCAAGCTGCCTT-GCCTGAGC-TACGAGACAGAGATCCTGACCGTGGAATACGGCCTGCTGCCTATCGGCAAGATCGTGGAAAAGCGGATCGAGTGCACCGTGTACAGCGTGGACAACAACGG-CAACATCTACACCCAGCCTGTGGCTCAGTGGCACGACAGAGGCAAGCAGAAGGTGTTCGAGTACTGCCTGGAAGATGGCAGCCTGATCAGAGCCACCAAGGAC-CACAAGTTCATGACAGTG-GACGGCCAGATGCTGCCCATCAAAGAGATCTTCCGGCGGAAGCTGGACCTGATGAGAGTGGACAACCTGCCTAATGGCTCTGGCGGCGAGAACCTGTACTTCCAAGGGGAAAATCTC-TACTTTCAAGGCGGCAGCGGCGGCATCGAGATCGCCACAGAGAAGTATCTGGGCGAGCAGAACGTGTACGACATCGGCGTGGAACGGGACCACAACTTCGCCCTGAAGAAC-GGCGGC-TACTTCCAGGGAATTGAGATTGCCACCGAGAAATACCTCGGGGAACAGAATGTGTATGA

	TATCGGAGTCGAGCGCGATCACAATTTTGCCCTGAAAAATGGCG-<br>GATCCCAGGTCCAGCTG-<br>CAAGAGTCTGGCGGAGGACTTGTTCAGGCTGGCGGAAGCCTGAGACTGAGCTGTACTGC<br>CAGCGGCAGAACCTTCAGCAGCTATGCCATGGGCTGGTTCAGACAGACCCCTGGCAAA-<br>GAAC-<br>GCGAGTTCGTGGCCGCCATTACATGGGGCGGAAGCACAACACTGTACGCCGACTCTGTG<br>AAGGGCAGATTCACCATGAGCCGGGACAACGCCAAGAACACCGTGTACCTG-<br>CAGATGAACAGCCTGAAGCCAGAGGACACCGCCGTGTACTATTGTGCCGCCGATGGCAG<br>CCAGTACAGAAGCACCTACAGCTTCAGAGACAAGCCCGACTACGGCTCTT-<br>GGGGCCAGGGAACACAAGTGACCGTGTCCTCTCACCACCACCATCACCATTGAATCTAG<br>ACCTTCTGCGGGGCTTGCCTTCTGGCCATGCCCTTCTTCTCTCCCTTGCACCTGTAC-<br>CTCTT-<br>GGTCTTTGAATAAAGCCTGAGTAGGAAAAAAAAAAAAAAAAAAAAAAAAAAAAAAAAAAA<br>AAAAAAAAAAAAAAAAAAAAAAAAAAAAAAAAAAAAAAAAAAAAAAAAAAAAAAAAAAA<br>AAAAAAAAAAAAAAAAAAAAAAAAAAAAAAAAAAAAA
ID	hBarnase-65C
Template pDNA	pUTR2-hBarnase-65C
Primers	HNC-542, HNT-7 and HNT-9
Features	T7 promoter (for CleanCap AG Reagent): 11-30, Kozak sequence (including start codon): 79-87, Anti-eDHFR nanobody CA1698 gene: 91-459, Caged eNpu C-intein gene: 466-792, C-terminal split Barnase gene: 793-933, Stop codon: 934-936
Sequence	CAGTGAATTGTAATACGACTCACTATAAGGCGAATTAAGAGAGAAAAGAAGAGTAA-<br>GAA-<br>GAAATATAAGACACCGGTCGCCACCATGGCCCAGGTCCAGCTGCAAGAGTCTGGCGGA<br>GGACTTGTTCAGGCTGGCGGAAGCCTGAGACTGAGCTGTAAAGCCAGCGG-<br>CATCATCTTCAGCGTGTACAAGATGACCTGGTACAGACAGGCCCCTGGCAAAGAGAG<br>AGCTGGTTGCCCTGATCACCACCAACAACAATACCATGACCGTGGACAGCGTGAAGGG-<br>CAGATTCACCATCAGCCGGGACAACGTGCAGAACACCGTGTACCTGGAAATGAACAAT<br>CTGAAGCCCGAGGACACCGCCGTGTACTACTGCAACGCCAATAGAGGACTGGCCGGAC-<br>CTGCCTATTGGGGCCAGGGAACACAAGTGACCGTGTCCTCTCACCACCACCATCACCAT<br>GGATCCGGCGAACAAGAAGTGTTCGAGTACTGCCTGGAAGATGG-<br>CAGCCTGATCAGAGCCAC-<br>CAAGGACCACAAGTTCATGACCGTGGACGGCCAGATGCTGCCCATCGACGAGATCTTCG<br>AGCGCGAGCTGGACCTGATGAGAGTGGACAACCTGCCTAATGGCTCTGGCGGCGA-<br>GAACCTG-<br>TACTTCCAAGGGGAAAATCTCTACTTTCAAGGCGGCAGCGGCGGCATCAAGATCGCCAC<br>AAGAAAGTACCTGGGCAAGCAGAACGTGTACGACATCGGCGTGGAACGGGAC-<br>CACAACTTCGCCCTGAAGAACGGCTTTATCGCCAGCAACTGCGGCAAGAGCGGCAGAA<br>CTTGGAGAGAGGCCGACATCAACTACACCAGCGGCTTCCGGAACAGCGACCG-<br>GATCCTG-<br>TACAGCAGCGACTGGCTGATCTACAAGACCACCGACCACTACCAGACCTTCACCAAGAT

	CAGATGAATCTAGACCTTCTGCGGGGCTTGCCTTCTGGCCATGCCCTTCTTCTCTCCCTT-<br>GCACCTGTACCTCTTGGTCTTTGAATAAAGCCTGAGTAGGAAAAAAAAAAAAAAAAAA<br>AAAAAAAAAAAAAAAAAAAAAAAAAAAAAAAAAAAAAAAAAAAAAAAAAAAAAA<br>AAAAAAAAAAAAAAAAAAAAAAAAAAAAAAAAAAAAAAAAAAAAAA
ID	hBarnase-81N
Template pDNA	pUTR2-hBarnase-81N
Primers	HNC-542, HNT-7 and HNT-9
Features	T7 promoter (for CleanCap AG Reagent): 11-30, Kozak sequence (including start codon): 79-87, N-terminal split Barnase gene: 88-327, Caged eNpu N-intein gene: 328-894, Anti-eDHFR nanobody Nb113 gene: 901-1302, Stop codon: 1303-1305
Sequence	CAGTGAATTGTAATACGACTCACTATAAGGCGAATTAAGAGAGAAAAGAAGAGTAA-<br>GAA-<br>GAAATATAAGACACCGGTCGCCACCATGGCCCAAGTGATCAACACCTTCGACGGCGTGG<br>CCGACTACCTGCAGACATACCACAAGCTGCCCGACAACTACATCACCAA-<br>GAGCGAGGCCCAGGCTCTCGGATGGGTTGCCTCTAAGGGAAACCTGGCCGATGTGGCCC<br>CTGGCAAGTCTATCGGCGGCGACATCTTCAGCAACAGAGAGGGCAAGCTGCCTGGCAA-<br>GAGCGGCAGAACTTGGAGAGAGGCCGACATCAACTACACCAGCTGCCTGAGCTACGAG<br>ACAGAGATCCTGACCGTGGAATACGGCCTGCTGCCTATCGGCAAGATCGTGGAAAA-<br>GCG-<br>GATCGAGTGCACCGTGTACAGCGTGGACAACAACGGCAACATCTACACCCAGCCTGTGG<br>CTCAGTGGCACGACAGAGGCAAGCAGAAGGTGTTCGAGTACTGCCTGGAAGATGG-<br>CAGCCTGATCAGAGCCACCAAGGACCACAAGTTCATGACAGTGGACGGCCAGATGCTG<br>CCCATCAAAGAGATCTTCCGGCGGAAGCTGGACCTGATGAGAGTGGACAAC-<br>CTGCCTAATGGCTCTGGCGGCGAGAACCTGTACTTCCAAGGGGAAAATCTCTACTTTCAA<br>GGCGGCAGCGGCGGCATCGAGATCGCCACAGAGAAGTATCTGGGCGAGCAGAACGTG-<br>TAC-<br>GACATCGGCGTGGAACGGGACCACAACTTCGCCCTGAAGAACGGCGGCTACTTCCAGG<br>GAATTGAGATTGCCACCGAGAAATACCTCGGGGAACAGAATGTGTATGATATCGGAG-<br>TCGAGCGCGATCACAATTTTGCCCTGAAAAATGGCGGATCCCAGGTCCAGCTGCAAGAG<br>TCTGGCGGAGGACTTGTTCAGGCTGGCGGAAGCCTGAGACTGAGCTGTACTGCCAGCGG-<br>CAGAACCTTCAGCAGCTATGCCATGGGCTGGTTCAGACAGACCCCTGGCAAAGAACGCG<br>AGTTCGTGGCCGCCATTACATGGGGCGGAAGCACAACACTGTAC-<br>GCCGACTCTGTGAAGGG-<br>CAGATTCACCATGAGCCGGGACAACGCCAAGAACACCGTGTACCTGCAGATGAACAGC<br>CTGAAGCCAGAGGACACCGCCGTGTACTATTGTGCCGCCGATGGCAGCCAGTACAGAA-<br>GCAC-<br>CTACAGCTTCAGAGACAAGCCCGACTACGGCTCTTGGGGCCAGGGAACACAAGTGACC<br>GTGTCCTCTCACCACCACCATCACCATTGAATCTAGACCTTCTGCGGGGCTT-<br>GCCTTCTGGCCATGCCCTTCTTCTCTCCCTTGCACCTGTACCTCTTGGTCTTTGAATAAAGC<br>CTGAGTAG-<br>GAAAAAAAAAAAAAAAAAAAAAAAAAAAAAAAAAAAAAAAAAAAAAAAAAAAAA

	AAAAAAAAAAAAAAAAAAAAAAAAAAAAAAAAAAAAAAAAAAAAAAAAAAAAAAA<br>AAAAAAAAAAA
ID	hBarnase-81C
Template pDNA	pUTR2-hBarnase-81C
Primers	HNC-542, HNT-7 and HNT-9
Features	T7 promoter (for CleanCap AG Reagent): 11-30, Kozak sequence (including start codon): 79-87, Anti-eDHFR nanobody CA1698 gene: 91-459, Caged eNpu C-intein gene: 466-792, C-terminal split Barnase gene: 793-885, Stop codon: 886-888
Sequence	CAGTGAATTGTAATACGACTCACTATAAGGCGAATTAAGAGAGAAAAGAAGAGTAA-<br>GAA-<br>GAAATATAAGACACCGGTCGCCACCATGGCCCAGGTCCAGCTGCAAGAGTCTGGCGGA<br>GGACTTGTTCAGGCTGGCGGAAGCCTGAGACTGAGCTGTAAAGCCAGCGG-<br>CATCATCTTCAGCGTGTACAAGATGACCTGGTACAGACAGGCCCCTGGCAAAGAGAGAG<br>AGCTGGTTGCCCTGATCACCACCAACAACAATACCATGACCGTGGACAGCGTGAAGGG-<br>CAGATTCACCATCAGCCGGGACAACGTGCAGAACACCGTGTACCTGGAAATGAACAAT<br>CTGAAGCCCGAGGACACCGCCGTGTACTACTGCAACGCCAATAGAGGACTGGCCGGAC-<br>CTGCCTATTGGGGCCAGGGAACACAAGTGACCGTGTCCTCTCACCACCACCATCACCAT<br>GGATCCGGCGAACAAGAAGTGTTCGAGTACTGCCTGGAAGATGG-<br>CAGCCTGATCAGAGCCAC-<br>CAAGGACCACAAGTTCATGACCGTGGACGGCCAGATGCTGCCCATCGACGAGATCTTCG<br>AGCGCGAGCTGGACCTGATGAGAGTGGACAACCTGCCTAATGGCTCTGGCGGCGA-<br>GAACCTG-<br>TACTTCCAAGGGGAAAATCTCTACTTTCAAGGCGGCAGCGGCGGCATCAAGATCGCCAC<br>AAGAAAGTACCTGGGCAAGCAGAACGTGTACGACATCGGCGTGGAACGGGAC-<br>CACAACTTCGCCCTGAAGAACGGCTTTATCGCCAGCAACTGCGGCTTCCGGAACAGCGA<br>CCGGATCCTGTACAGCAGCGACTGGCTGATCTACAAGACCACCGACCACTACCAGAC-<br>CTTCACCAAGATCAGATGAATCTAGACCTTCTGCGGGGCTTGCCTTCTGGCCATGCCCTT<br>CTTCTCTCCCTTGCACCTGTACCTCTTGGTCTTTGAATAAAGCCTGAGTAG-<br>GAAAAAAAAAAAAAAAAAAAAAAAAAAAAAAAAAAAAAAAAAAAAAAAAAAAAAAA<br>AAAAAAAAAAAAAAAAAAAAAAAAAAAAAAAAAAAAAAAAAAAAAAAAAAAAAAA<br>AAAAAAAAAAA
ID	hBarnase-81N-Lag16
Template pDNA	pUTR2-hBarnase-81N-eNpuNcage-Lag16
Primers	HNC-542, HNT-7 and HNT-9
Features	T7 promoter (for CleanCap AG Reagent): 11-30, Kozak sequence (including start codon): 79-87, N-terminal split Barnase gene: 88-327, Caged eNpu N-intein gene: 328-894, Anti-EGFP Lag16 nanobody gene: 901-1281, Stop codon: 1282-1284
Sequence	CAGTGAATTGTAATACGACTCACTATAAGGCGAATTAAGAGAGAAAAGAAGAGTAA-<br>GAA-<br>GAAATATAAGACACCGGTCGCCACCATGGCCCAAGTGATCAACACCTTCGACGGCGTGG

	CCGACTACCTGCAGACATACCACAAGCTGCCCGACAACTACATCACCAA-<br>GAGCGAGGCCCAGGCTCTCGGATGGGTTGCCTCTAAGGGAAACCTGGCCGATGTGGCCC<br>CTGGCAAGTCTATCGGCGGCGACATCTTCAGCAACAGAGAGGGCAAGCTGCCTGGCAA-<br>GAGCGGCAGAACTTGGAGAGAGGCCGACATCAACTACACCAGCTGCCTGAGCTACGAG<br>ACAGAGATCCTGACCGTGGAATACGGCCTGCTGCCTATCGGCAAGATCGTGGAAAA-<br>GCG-<br>GATCGAGTGCACCGTGTACAGCGTGGACAACAACGGCAACATCTACACCCAGCCTGTGG<br>CTCAGTGGCACGACAGAGGCAAGCAGAAGGTGTTCGAGTACTGCCTGGAAGATGG-<br>CAGCCTGATCAGAGCCACCAAGGACCACAAGTTCATGACAGTGGACGGCCAGATGCTG<br>CCCATCAAAGAGATCTTCCGGCGGAAGCTGGACCTGATGAGAGTGGACAAC-<br>CTGCCTAATGGCTCTGGCGGCGAGAACCTGTACTTCCAAGGGGAAAATCTCTACTTTCAA<br>GGCGGCAGCGGCGGCATCGAGATCGCCACAGAGAAGTATCTGGGCGAGCAGAACGTG-<br>TAC-<br>GACATCGGCGTGGAACGGGACCACAACTTCGCCCTGAAGAACGGCGGCTACTTCCAGG<br>GAATTGAGATTGCCACCGAGAAATACCTCGGGGAACAGAATGTGTATGATATCGGAG-<br>TCGAGCGCGATCACAATTTTGCCCTGAAAAATGGCGGATCCGCTCAGGTGCAGCTGGTG<br>GAATCTGGTGGTAGACTGGTGCAGGCCGGCGATAGCCTGAGACTGTCTT-<br>GTGCCGCCAGCGG-<br>CAGAACCTTTAGCACATCTGCCATGGCCTGGTTCAGACAGGCCCCTGGCAGAGAAAGGG<br>AATTCGTGGCCGCCATCACATGGACCGTGGGCAATAC-<br>CATCCTGGGCGACTCTGTGAAGGG-<br>CAGATTCACCATCAGCCGGGACAGAGCCAAGAACACCGTGGACCTGCAGATGGACAAC<br>CTGGAACCTGAGGACACCGCCGTGTACTACTGCAGCGCCAGATCTAGAGGCTAC-<br>GTGCTGTCCGTGCTGAGAAGCGTGGACAGCTACGATTATTGGGGCCAGGGCACCCAAGT<br>GACCGTGTCTTGAATCTAGACCTTCTGCGGGGCTT-<br>GCCTTCTGGCCATGCCCTTCTTCTCTCCCTTGCACCTGTACCTCTTGGTCTTTGAATAAAGC<br>CTGAGTAG-<br>GAAAAAAAAAAAAAAAAAAAAAAAAAAAAAAAAAAAAAAAAAAAAAAAAAAA<br>AAAAAAAAAAAAAAAAAAAAAAAAAAAAAAAAAAAAAAAAAAAAAAAAAAAA<br>AAAAAAAAAAA
ID	GFPenhNb-hBarnase-81C
Template pDNA	pUTR2-GFPenhNb-NpuCcage-hBarnase-81C
Primers	HNC-542, HNT-7 and HNT-9
Features	T7 promoter (for CleanCap AG Reagent): 11-30, Kozak sequence (including start codon): 79-87, Anti-EFGP GFP enhancer nanobody gene: 88-432, Caged eNpu C-intein gene: 439-765, C-terminal split Barnase gene: 766-858, Stop codon: 859-861
Sequence	CAGTGAATTGTAATACGACTCACTATAAGGCGAATTAAGAGAGAAAAGAAGAGTAA-<br>GAA-<br>GAAATATAAGACACCGGTCGCCACCATGGCTCAGGTGCAGCTTGTTGAATCTGGCGGAG

	CACTGGTTCAGCCTGGCGGATCTCTGAGACTGTCTT-GTGCCGCCTCTGGCTTCCCCGTGAAC-CGGTACAGCATGCGGTGGTATAGACAGGCCCCTGGCAAAGAACGAGAGTGGGTGGCCGGAATGTCTAGCGCTGGCGATAGAAGCAGCTACGAGGACAGCGTGAAGGGCAGATTCAC-CATCAGCAGGGACGACGCCAGAAACACCGTGTACCTGCAGATGAACAGCCTGAAGCCTGAGGACACCGCCGTGTACTACTGCAACGTGAACGTGGGCTTCGAG-TACTGGGGCCAGGGAACCCAAGTGACCGTTTCTGGATCCGGCGAACAAGAAGTGTTCGAGTACTGCCTGGAAGATGGCAGCCTGATCAGAGCCACCAAGGACCACAAGTTCATGAC-CGTG-GACGGCCAGATGCTGCCCATCGACGAGATCTTCGAGCGCGAGCTGGACCTGATGAGAGTGGACAACCTGCCTAATGGCTCTGGCGGCGAGAACCTGTACTTCCAAGGGGAAAATCTC-TACTTTCAAGGCGGCAGCGGCGGCATCAAGATCGCCACAAGAAAGTACCTGGGCAAGCAGAACGTGTACGACATCGGCGTGGAACGGGACCACAACTTCGCCCTGAAGAAC-GGCTTTATCGCCAGCAACTGCGGCTTCCGGAACAGCGACCGGATCCTGTACAGCAGCGACTGGCTGATCTACAAGACCACCGACCACTACCAGACCTTCACCAA-GATCAGATGAATCTA-GACCTTCTGCGGGGCTTGCCTTCTGGCCATGCCCTTCTTCTCTCCCTTGCACCTGTACCTCTTGGTCTTTGAATAAAGCCTGAGTAG-GAAAAAAAAAAAAAAAAAAAAAAAAAAAAAAAAAAAAAAAAAAAAAAAAAAAAAAAAAAAAAAAAAAAAAAAAAAAAAAAAAAAAAAAAAAAAAAAAAAAAAAAAAAAAAAAAAAAAAAA
ID	hBarstar
Template pDNA	pUTR2-hBarstar
Primers	HNC-542, HNT-7 and HNT-9
Features	T7 promoter (for CleanCap AG Reagent): 11-30, Kozak sequence (including start codon): 79-87, hBarstar Gene: 88-357, Stop Codon: 358-360
Sequence	CAGTGAATTGTAATACGACTCACTATAAGGCGAATTAAGAGAGAAAAGAAGAGTAA-GAA-GAAATATAAGACACCGGTCGCCACCATGGCCAAGAAAGCCGTGATCAACGGCGAGCAGATCCGGTCCATCAGCGACCTGCACCAGACACTGAAGAAAGAGCTGGCCCTGCCTGAG-TACTACGGCGAGAATCTGGATGCCCTGTGGGATTGTCTGACCGGCTGGGTCGAATACCCTCTGGTTCTGGAATGGCGGCAGTTCGAGCAGAGCAAGCAGCTGACAGA-GAATGGCGCCGA-GAGCGTGCTCCAGGTGTTCAGAGAAGCCAAGGCCGAGGGCTGCGACATCACCATCATCCTGAGCTGAATCTAGACCTTCTGCGGGGCTTGCCTTCTGGCCATGCCCTTCTTCTCTCCCTT-GCACCTGTACCTCTTGGTCTTTGAATAAAGCCTGAGTAGGAAAAAAAAAAAAAAAAAAAAAAAAAAAAAAAAAAAAAAAAAAAAAAAAAAAAAAAAAAAAAAAAAAAAAAAAAAAAAAAAAAAAAAAAAAAAAAAAAAAAAAAAAAAAAAAAAAAA
